# Supplementary material for: BMP-Dependent Mobilization of Fatty Acid Metabolism Promotes Caenorhabditis elegans Survival on a Bacterial Pathogen
Source: bioRxiv. 2025 Mar 15:2025.03.13.643118. Preprint. [Version 1] doi: 10.1101/2025.03.13.643118 (PMC11952492; doi:10.1101/2025.03.13.643118)
Supplement: 1 [file NIHPP2025.03.13.643118V1-supplement-1.pdf]

bioRxiv preprint doi: https://doi.org/10.1101/2025.03.13.643118; this version posted March 15, 2025. The copyright holder for this preprint (which was not certified by peer review) is the author/funder, who has granted bioRxiv a license to display the preprint in perpetuity. It is made available under aCC-BY 4.0 International license.

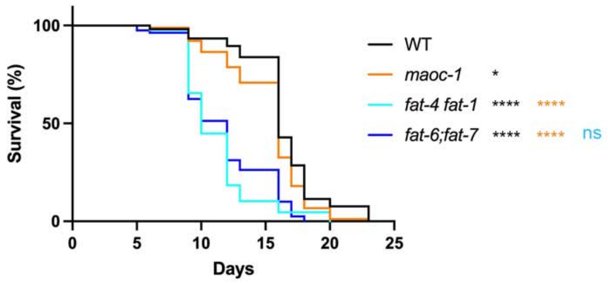

Figure S1. Fatty acid desaturation is more involved in survival after pathogen exposure than β-oxidation.

Survival analysis of wildtype, *maoc-1*, *fat-4 fat-1* and *fat-6;fat-7* animals on *S. marcescens* bacteria. N values: WT (105), *maoc-1* (89), *fat-4 fat-1* (87), *fat-6;fat-7* (80).

ns = p > 0.01; * = p<=0.05; ** = p<=0.01; *** = p<=0.001; **** = p<=0.0001.

Black asterisks denote significance relative to wildtype control; orange is significance relative to *maoc-1*; teal is significance relative to *fat-4 fat-1*.
